# NO-dependent attenuation of TPA-induced immunoinflammatory skin changes in Balb/c mice by pindolol, heptaminol or ATRA, but not by verapamil

**DOI:** 10.18632/oncotarget.10217

**Published:** 2016-06-22

**Authors:** Jinhyuk F. Chung, Calvin J. Yoon, Seon Ah Cheon, Eun Seok Seo, Sung Ho Park, Jae Seung Yang, Bumju Kim, Min Young Joo, Tae Jung Park, Ki Hean Kim, Anil K. Sood, Sang Joon Lee

**Affiliations:** ^1^ Synergy Point Co., Sungnam, South Korea; ^2^ Division of Integrative Biosciences and Biotechnology (IBB), Pohang University of Science and Technology (POSTECH), Pohang, South Korea; ^3^ Center for Biofluid and Biomimic Research, Department of Mechanical Engineering, Pohang University of Science and Technology (POSTECH), Pohang, South Korea; ^4^ NanoBio-Chemistry Laboratory, Department of Chemistry, Chung-Ang University, Seoul, South Korea; ^5^ Clinical Immunology, Laboratory Science Unit, International Vaccine Institute, Seoul, South Korea; ^6^ Departments of Gynecologic Oncology and Reproductive Medicine and Cancer Biology, The University of Texas MD Anderson Cancer Center, Houston, TX, USA

**Keywords:** nitric oxide, beta-blocker, heptaminol, tumor promotion, phorbol

## Abstract

Recently a mouse skin carcinogenesis study reported that a β-blocker carvedilol displayed antitumor-properties via antihyperplastic effects. However, the antihyperplastic mechanism is unclear as the β-blocker is characterized with multiple pleiotropic effects including stimulation of endothelial NO release and verapamil-like calcium channel blocking activity. To investigate the nature and the origin of the antihyperplastic effects, we tested topical pretreatment with pindolol, heptaminol, ATRA or verapamil against Balb/c mouse ear skin hyperplasia that was induced by TPA. We found that pindolol, heptaminol or ATRA, but not verapamil, inhibited the TPA-induced immunoinflammatory skin changes in an NO-dependent manner, which included epidermal hyperplasia, skin edema and fibrosis. Furthermore, we also observed NO-dependent alleviation of the TPA-induced NK cell depletion in the ear tissues by heptaminol pretreatment. Together our results suggest that stimulation of NO generation from constitutive synthases may be primarily responsible for the reported antihyperplastic and NK cell-preserving effects of the β-blockers, and that similar effects may be observed in other immunity normalizing compounds that also promote endothelial NO synthesis.

## INTRODUCTION

Chronic neuropsychological stress causing the activation of β-adrenergic receptors leads to increased rate of metastasis and tumor recurrence by inhibiting normal immune functions in cancer patients [1, 2]. Based on this idea, Powe et al. reported that long-term use of general β-adrenergic receptor inhibitors (β-blockers) was associated with reduced incidence of metastasis and secondary tumor formation among breast cancer patients [2], while later studies reported that only certain subsets of β-blockers were clinically effective in cancer treatments. In a multicenter retrospective study, Watkins et al. reported that the use of non-selective β-blockers during chemotherapy was characterized with marked increase in median overall survival (OS) when compared with selective β-blocker use or non-use (OS 94.9 months vs. 38 months vs. 42 months, respectively) [3]. More specifically, retrospective and prospective clinical studies have demonstrated that the use of a first generation non-specific β-blocker propranolol, but not atenolol, is associated with improved treatment outcome in cancer patients via suppression of distant metastasis and relapse without any effects on the growth of primary tumors [4, 5]. Furthermore, similar clinical benefits have not been reported on other β-blockers with exception to carvedilol, which was associated with significantly reduced risk of cancer at all sites (hazard ratio (HR) 0.74, 95% confidence interval (CI): 0.63-0.87, *p* < 0.001) with particularly strong risk reduction observed against stomach (HR 0.30: 0.14-0.63) and lung (HR 0.59: 0.37–0.94) cancers [6]. These observations collectively suggest that the clinical benefits of using β-blockers in cancer treatments may not be generalized to other β-blockers, and hence understanding the responsible mechanism is critical in identification of the optimal β-blockers for adjuvant cancer therapeutic use.

In support of this view, preclinical studies have demonstrated that antitumor and chemopotentiating effects of β-blockers are limited to certain compounds, which could not be generalized by their β-adrenoreceptor selectivity as widely contrasting *in vitro* antitumor effects were reported between α1,β1,β2-blockers carvedilol and labetalol, and between selective β1-blockers nebivolol and atenolol [7]. Furthermore, a recent chemical mouse skin carcinogenesis study on immunocompetent SENCAR mice demonstrated that oral or topical carvedilol, but not atenolol, exerted antitumor-promoting activity by suppressing the skin inflammation and epidermal hyperplasia [8]. Also, in an animal study modeling the pulmonary metastatic effects of surgery stress using immunocompetent F344 rats and MADB106 breast cancer cell line, non-specific β-blocker nadolol was shown to exert its antimetastitic effects in lungs by attenuating the stress-induced reduction in pulmonary-marginating natural killer cell (NK cell) numbers and individual NK cell activity [9]. Given the observations, it was recently suggested that the immunoinflammation-modulating pleiotropic effects of propranolol and carvedilol might be important contributing mechanisms toward their reported cancer therapeutic benefits, which included their endothelial nitric oxide (NO) release stimulation and verapamil-like calcium channel blocking activity (CCB) [10]. Therefore, we decided to assess the contribution of the two pleiotropic effects of β-blockers in modulating a model tumor-promoting inflammation by 12-O-tetradecanoylphorbol-13-acetate (TPA).

In present study, we studied immunoinflammation-modulating effects of topical pindolol, heptaminol, and verapamil in Balb/c mouse ear skin against the acute inflammation induced by the potent tumor-promoting agent TPA, and involvement of NO in the process. Pretreatment effect of all-trans retinoic acid (ATRA), a known inhibitor of TPA-induced skin tumor promotion, was also studied for comparison. Briefly, pindolol is a non-specific β-blocker with partial β3 agonist activity and potent endothelial NO-inducing capacity [11]. Pindolol was chosen over carvedilol for current study due to its lack of α-adrenoreceptor blocking activity. Heptaminol, on the other hand, is a vasodilator and a general antagonist to catecholamine release and uptake that also raises intracellular free calcium level [12, 13]. Lastly verapamil is an L-type calcium channel blocker.

## RESULTS

### Topical pretreatment with ATRA, pindolol or heptaminol, but not verapamil, markedly attenuates acute TPA-induced epidermal hyperplasia, edema and fibrosis in an NO-dependent manner

**Figure 1:**
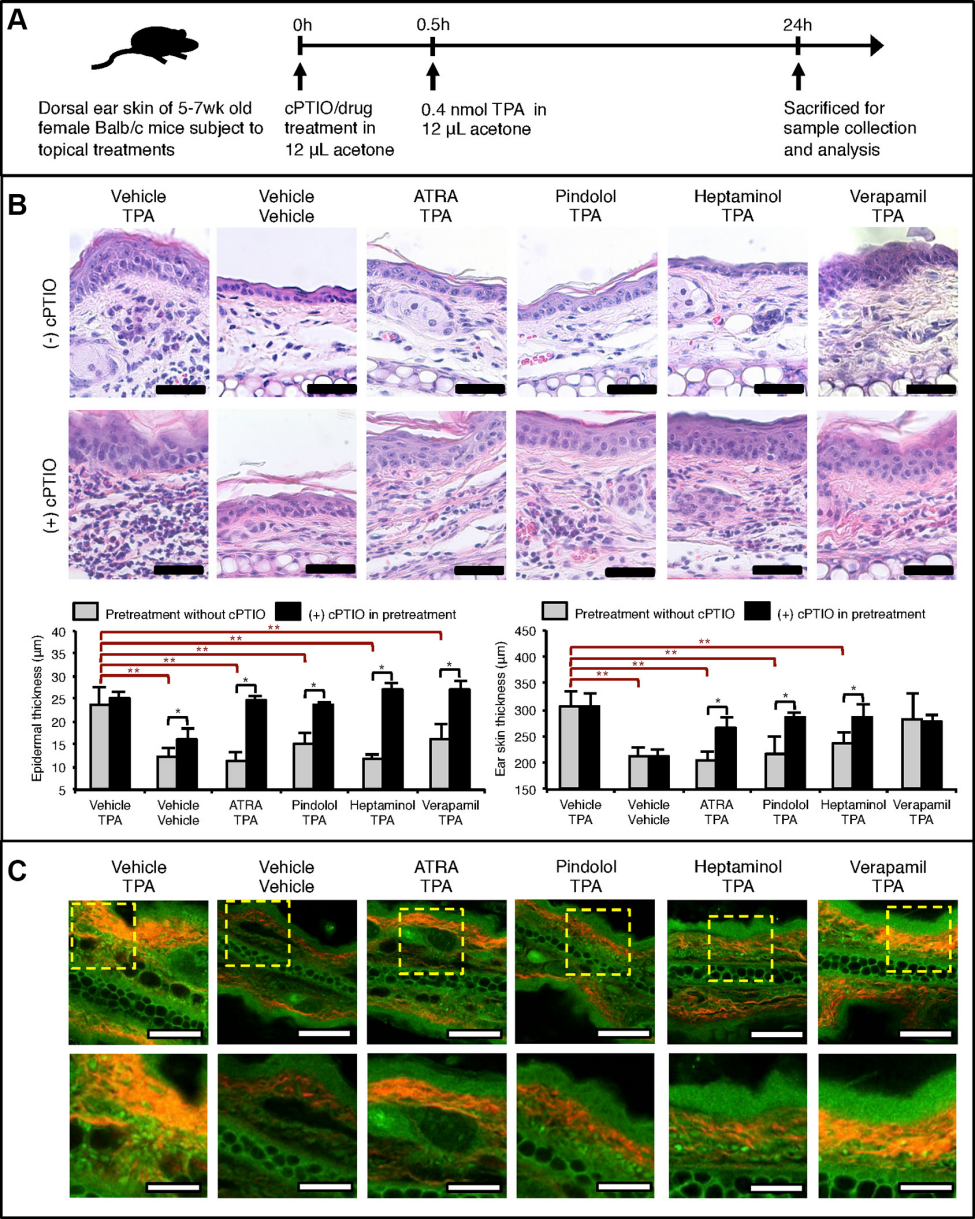
NO-dependent drug pretreatment effects against acute TPA-induced inflammatory skin changes in Balb/c mouse ear skin (**A**) Graphical outline of topical drug and TPA treatment schedule prior to the skin sample analysis. All treatments were topically applied using 12 μL acetone as the vehicle with exception to heptaminol, which was dissolved in 50% water/acetone. Following doses of each compound per mouse ear was applied: TPA = 0.4 nmol, cPTIO = 1 nmol, ATRA = 1 nmol, pindolol = 2 nmol, heptaminol = 2.5 nmol and verapamil = 2.2 nmol. (**B**) Histological analysis on the collected mouse ears after H&E-staining. TOP: Representative images of the stained tissue slides showing NO-dependent attenuation of the TPA-induced cellular changes, epidermal hyperplasia, fibrosis, and skin inflammation by ATRA, pindolol, or heptaminol pretreatments. Scale bar = 50 μm. BOTTOM: Quantified changes in the epidermal thickness and ear skin thickness in each treatment groups. Asterisk (*) denotes drug pretreatment groups whose protective effects were abolished by the addition of cPTIO in the pretreatment step (*p* < 0.05). Double asterisks (**) denote significant protective effects from drug pretreatment effects in the absence of cPTIO (*p* < 0.05). Averages were calculated from 3 averaged specimen readings from 3 biological replicates (*n* = 9). (**C**) Representative images of the same tissue slides observed under SHG-augmented TPM showing reduced fibrosis in the ear specimens pretreated with ATRA, pindolol or heptaminol, but not verapamil. Red color notes SHG signal from collagen fibers. Also, highly autofluorscent granular cells were noted in the dermis of every TPA-exposed specimen except those pretreated with ATRA. TOP: 20× magnified view. Scale bar = 100 μm. BOTTOM: Zoom-in of the dotted box. Scale bar = 40 μm.

Some of the key tumor-promoting features of TPA-exposed mouse skin include hyperplastic changes in the epidermis, inflammatory dermal swelling and activation of fibroblasts with resulting fibrosis. Particularly, TPA-induced activation of fibroblasts is critical during the tumor promotion process as they maintain the chronic inflammation state by attracting macrophages and neutrophils through high-level secretion of monocyte chemotactic protein-1 (MCP1) [14, 15]. Therefore, we first assessed histological changes after 24 hours in the acute TPA-exposed mouse ear skin, subject to 30 min pretreatment with ATRA, pindolol, heptaminol or verapamil (Figure 1A). Briefly, the dose of TPA used in the experiment at 0.4 nmol in 12 μL acetone is approximately equivalent per skin surface area to a mildly tumor promoting dose at 5 nmol in 100 μL acetone due to the smaller surface area of mouse ear skin at 0.785 cm^2^. This adjustment was determined by a preliminary experiment that assessed the surface area coverage by 100 μL acetone containing rhodamine to be 5.5 cm^2^. Also, through a series of preliminary studies, we experimentally determined the minimum dose of ATRA, pindolol and heptaminol that significantly attenuated the TPA-induced edema (*p* < 0.05) to be at 1 nmol, 2 nmol, and 2.5 nmol, respectively, via 12 μL vehicle per mouse ear (Figure 1B). Verapamil did not attenuate the TPA-induced swelling at 2.2 nmol per mouse ear (Figure 1B), and in confirmation of its lack of protective effects, increasing the dose up to 11 nmol per mouse ear further increased the tissue swelling, epidermal hyperplasia and inflammatory damages to the skin tissues (Supplementary Figure S1).

Close inspection of the epidermis revealed that all pretreatments, including those with verapamil, significantly attenuated the TPA-induced epidermal hyperplasia (*p* < 0.05), although verapamil pretreatment failed to protect against the atypical cellular changes in the epidermal cells (Figure 1B: TOP). Additionally, while the ear skin samples pretreated with ATRA, pindolol or heptaminol were not characterized with signs of fibrosis, TPA-alone and verapamil-pretreated samples were frequently characterized with fibrosis-type dermal changes. Addition of 1 nmol soluble NO-scavenger carboxy-PTIO (cPTIO) during the pretreatment step severely aggravated the inflammation caused by TPA, while it also completely abolished the antihyperplastic, antiinflammatory and antifibrotic effects of all pretreatments tested (Figure 1B: BOTTOM). Interestingly, pretreatment with cPTIO alone was also sufficient in inducing mild epidermal hyperplasia, even in the absence of TPA treatment. Collectively these findings suggest that the antiinflammatory and antihyperplastic effects of ATRA, pindolol, or heptaminol pretreatments are commonly mediated by NO, whose endogenous availability also affords partial protective effects against the TPA-exposure.

Examination of the same ear specimens under two-photon microscopy (TPM) augmented with second harmonic generation (SHG) microscopy revealed additional details about the antiinflammatory effects of ATRA, pindolol and heptaminol against TPA. Collagen fibers produced by activated fibroblasts show strong SHG signals for imaging [16]. In this regard, marked attenuation of the SHG signal intensities and its emitting bodies in the ATRA, pindolol or heptaminol but not verapamil pretreated samples showing red fibrous structures (Figure 1C: TOP) confirmed the histological observation that ATRA, pindolol or heptaminol, but not verapamil inhibited the TPA-induced fibrosis. Also, close inspection of the specimens revealed a number of highly autofluorescent granular cells in the dermis of all TPA-treated mouse ears, which were not observed in the vehicle-treated ears or in those pretreated with ATRA (Figure 1C: BOTTOM). As activated neutrophils or macrophages that drive the tumor-promoting chronic inflammation by TPA [14, 17] emit strong NAD(P)H autofluorescence signal that can be readily identified on TPM with 710 nm excitation [18, 19], the observations of highly autofluorescent granular cells raised a possibility that the TPA-induced dermal uptake of neutrophils or macrophages might not be inhibited by the pretreatment with pindolol or heptaminol despite their net antihyperplastic and antiinflammatory effects (Figure 1B). Therefore, we decided to perform non-labeled autofluorescence TPM (afTPM) on live mouse ears for confirmation.

### afTPM on live mouse ears confirms that ATRA blocks the TPA-mediated vascular leakage and uptake of NAD(P)H-rich autofluorescent immune cells, while pindolol, heptaminol or verapamil do not

**Figure 2:**
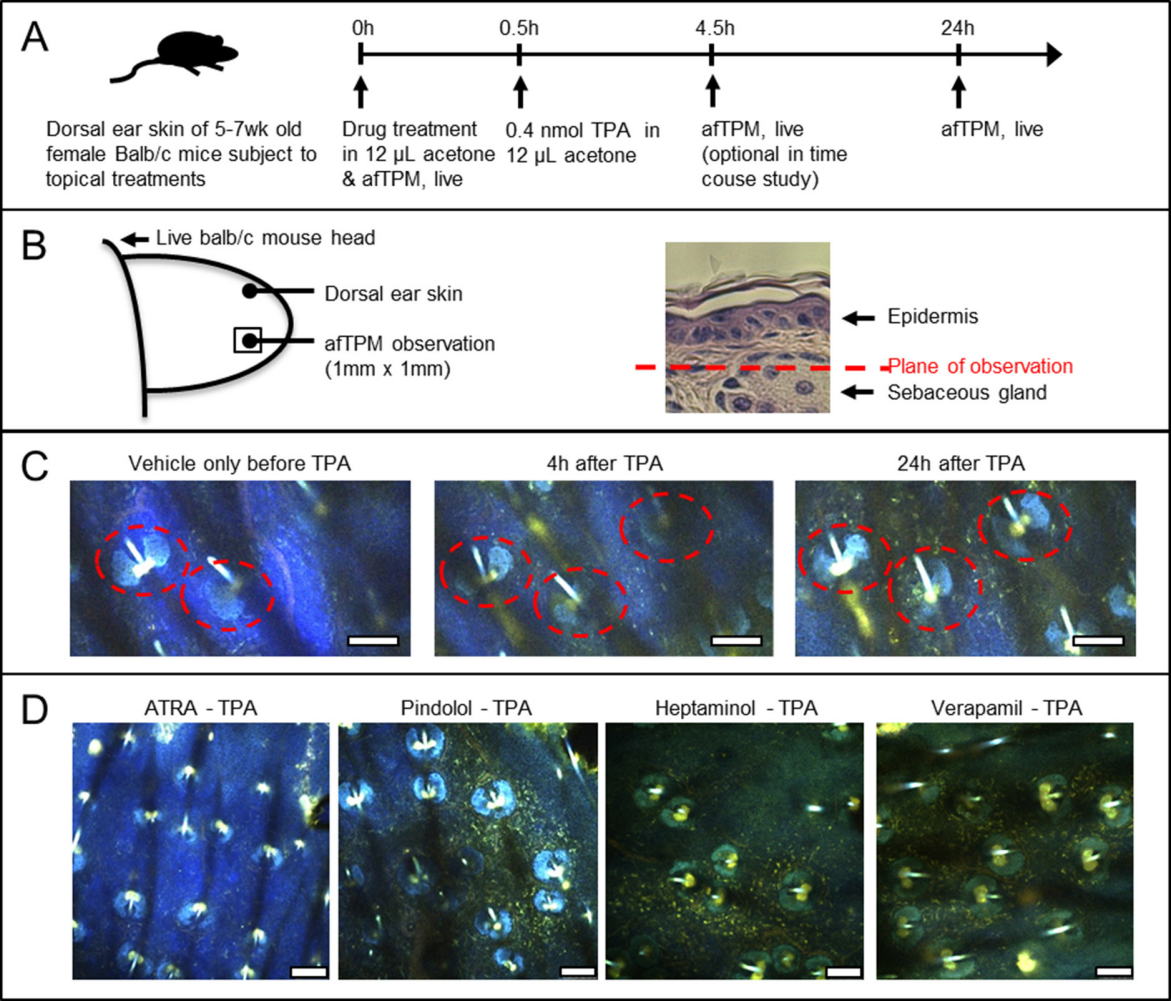
Confirmation by afTPM on the treated ears of live animals that only ATRA pretreatment, and no other agents, blocked the TPA-induced uptake of highly-autofluorescent leukocytes (**A**) Graphical outline of the afTPM experiment protocol on live Balb/c mouse ears. (**B**) Graphical representation of the ear tissue region that was characterized during afTPM procedure. (**C**) Time-resolved observation of inflammation development upon topical TPA treatment showing gradual migration of autofluorescent leukocytes and background tissue autofluoresence development. Dotted line notes sebaceous gland. Scale bar = 100 μm. (**D**) afTPM characterization of the treated live mouse ears 24 hours after the TPA application clearly showing marked accumulation of autofluorescent leukocytes and tissue autofluorescence development in pindolol, heptaminol or verapamil-pretreated ears against TPA. Pretreatment with ATRA, on the other hand, completely blocked the TPA-induced development of such changes. Scale bar = 100 μm.

afTPM on live mouse ear skin was previously demonstrated as a sensitive and convenient tool to characterize lipopolysaccharide (LPS)-induced vascular leakage and activated neutrophil/macrophage uptake in real-time due to their associated autofluorescence development [18, 20]. Based on the previously reported methodology of non-labeled afTPM on the dorsal skin of mouse ear [20], we decided to characterize the inflammatory effects of the topical TPA application and its attenuation by drug pretreatment (Figure 2A, 2B). Similar to the LPS-induced ear skin inflammation, time-lapse afTPM showed migration of autofluorescent leukocytes from capillaries to dermis as well general development of background autofluorescence that started becoming evident 4 hours after the topical TPA application and saturated after 24 hours (Figure 2C). Consistent with known TPA-effects and our histological findings (Figure 1B, 1C), these observations suggested that the topical TPA treatment caused uptake of neutrophils/macrophages and vascular leakage. Also consistent with the histological findings (Figure 1B, 1C), pretreatment with ATRA, but not pindolol, heptaminol or verapamil, completely blocked the development of background autofluorescence and uptake of autofluorescent leukocytes (Figure 2D). These findings consistently confirmed that the pretreatments with pindolol or heptaminol did not inhibit the TPA-induced uptake of neutrophils/macrophages, despite their net antihyperplastic and antiinflammatory effects against TPA in histological observations (Figures 1B, 2D).

### Topical pretreatment with heptaminol attenuates the TPA-induced depletion of marginating NK cells in the mouse ears and L-NAME abrogates this effect

**Figure 3:**
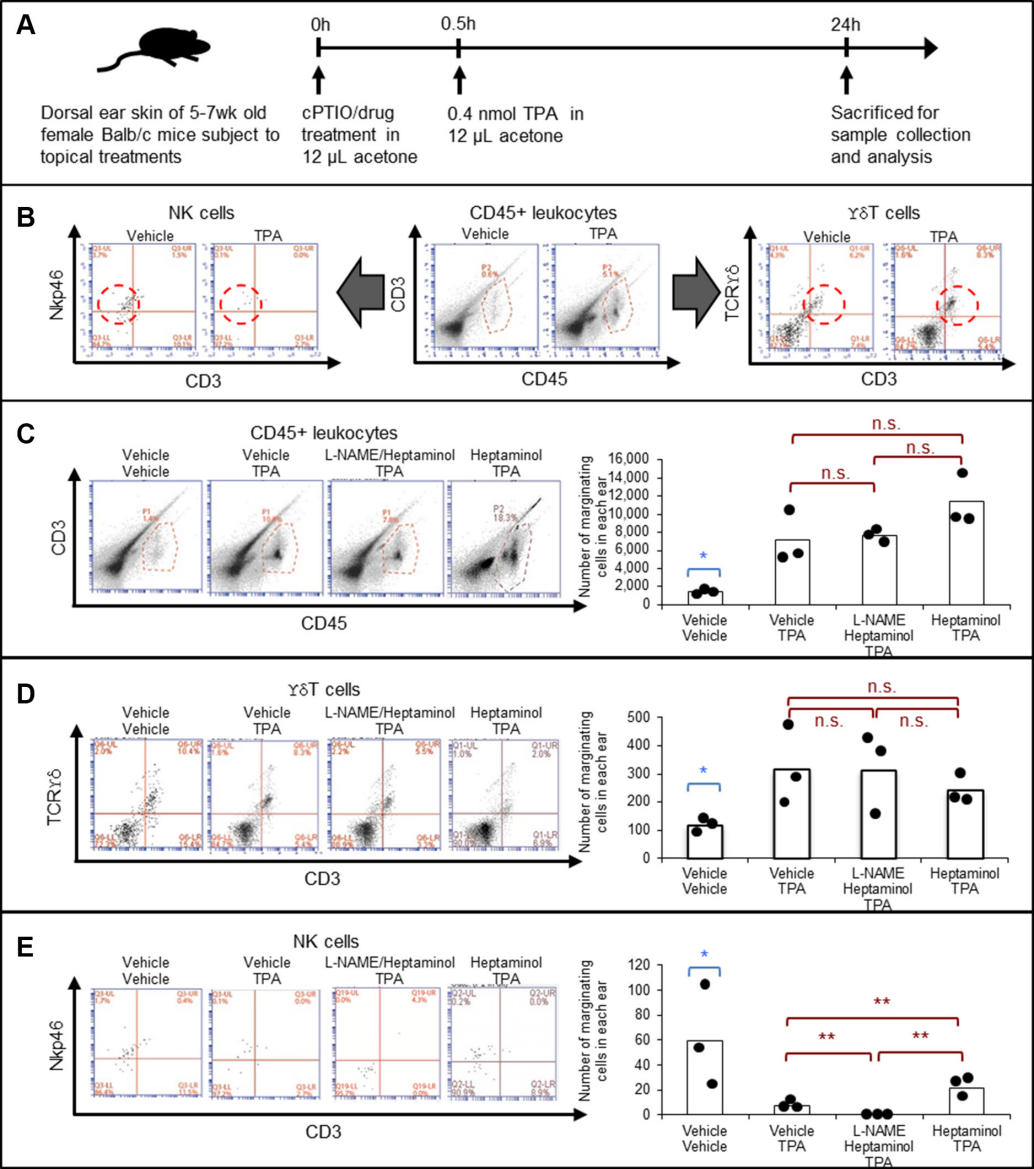
Heptaminol attenuates the TPA-induced selective depletion of tissue-marginating NK cells, and topical L-NAME pretreatment abrogates this protective effect (**A**) Graphical outline of the drug pretreatment protocol prior to the flow cytometric analyses on tissue-marginating immune cells in the mouse ears. (**B**) Gating strategy for identifying CD45+ leukocytes, ϒδ T cells and NK cells. Dotted lines indicate the target cells. (**C**) Quantification of the tissue-marginating CD45+ leukocytes in the treated ears. (**D**) Quantification of the tissue-marginating ϒδ T cells in the treated ears. (**E**) Quantification of the tissue-marginating NK cells in the treated ears. Asterisk (*) denotes significant difference between vehicle-treated control samples versus all other drug-treated specimens (*p* < 0.05). Double asterisks (**) denote significant difference between the two groups in comparison (*p* < 0.05). n.s.: not significant. Biological replication from three animals, *n* = 3.

While the antihyperplastic and antiinflammatory effects of pindolol or heptaminol pretreatment against TPA are potentially important antitumor-promoting features, normalization of peripheral NK cell availability against its depressing effects by stress was previously demonstrated as a key mechanism behind the immunopotentiating and antimetastatic properties of β-blockers against cancer in animal model studies [9, 21]. Therefore, we investigated the pretreatment effects of the catecholamine antagonist heptaminol against acute TPA effects on the local availability of marginating CD45+ leukocytes, ϒδ T cells and NK cells (Figure 3A) [22]. Briefly, dermal accumulation of CD45+ leukocytes is a hallmark feature of TPA-induced skin inflammation, while ϒδ T cells and NK cells play proximal roles in skin immune surveillance against cancer [23].

Consistent with conventional knowledge and current experimental data (Figures 1B, 2C, 2D), flow cytometry analysis on a mouse ear showed that TPA-treatment caused near 4-fold accumulation of CD45+ leukocytes, which on contrary, was not attenuated by pretreatment with heptaminol (Figure 3C). Quantification of ϒδ T cells showed similar results, whereby the pretreatment with heptaminol did not attenuate the 3-fold increase in the marginating ϒδ T cells from TPA-exposure (Figure 3D). In contrast, pretreatment with heptaminol attenuated TPA-induced depletion of the tissue-marginating NK cells in the mouse ear skin (40% of control vs. 15% of control, *p* < 0.05), although this attenuating effect was completely abolished when constitutive NOS (NOS1 + NOS3) inhibitor L-NAME was added during drug pretreatment step (Figure 3E; *p* < 0.01). This finding maybe explained by the fact that endogenous NO from NOS3 prevents NK cells from activation-induced self-apoptosis via suppression of TNF-α [24]. Alternatively, hemodynamic depletion of the marginating NK cells in the cPTIO/TPA-exposed skin from nitric oxide depletion and CD45+ leukocyte crowding is also possible since NO is needed in NK cell activation (For a review, see ref. [25]). Together, these results suggested that TPA-exposure selectively depleted the tissue-marginating NK cells, and that heptaminol pretreatment attenuated this effect via stimulation of NO synthesis by constitutive NOS.

## DISCUSSION

In the present study, we experimentally demonstrated that pretreatment with ATRA, pindolol or heptaminol, but not verapamil, significantly attenuated nearly all hyperplastic and inflammatory effects of an acute TPA treatment in female Balb/c mouse ear skin, and that these pretreatment effects were completely abolished by the addition of a soluble NO scavenger cPTIO (Figure 1B, 1C). These observations intuitively suggest that stimulation of NO generation by ATRA, pindolol, or heptaminol pretreatments is directly responsible for their attenuating effects against the tumor-promoting inflammation by TPA, and that respective agent's main pharmacological activity did not account for the observed attenuating effects (Figure 1B, 1C, Supplementary Figure S1). Consistent with this view, topical application of a direct NO donor nitroglycerin was previously demonstrated to attenuate TPA-induced epidermal hyperplasia and its subsequent tumor-promotion effect in 7,12-dimethylbenz(a)anthracene (DMBA)-initiated Swiss albino mouse skin. [26] Given the pivotal role of inducible NO synthase (NOS2) activation during the TPA-induced skin inflammation [27], the observed effects of ATRA, pindolol and heptaminol are likely to be mediated by downstream activation of constitutive NO synthases such as NOS1 or NOS3, although evidence of current study is insufficient in determining their respective contributions. However, it is worth noting that mouse ear skin is densely populated with microvessels that mainly express NOS3, in addition to the fact that skin fibroblasts were reported with antihyperplastic NO production from exclusively NOS3 upon exposure to β3-adrenoreceptor agonists [28].

While the present findings and those by others implicate stimulation of NO generation from constitutive synthases (mainly NOS3) as the main driver of antihyperplastic and antiinflammatory effects against the TPA-induced tumor-promoting tissue changes (Figure 1) [26], stimulated generation of NO from NOS3 or exogenous sources have been also reported with three other modes of direct and indirect anticancer mechanisms. Firstly, endogenous NO from NOS3 maintains lytic capacity of NK cells while it also protects NK cells from their activation-induced self-apoptosis via suppression of TNF-α [24]. This is consistent with our own finding that the addition of L-NAME abolished the attenuating effect of heptaminol against the depletion of tissue-marginating NK cell in the TPA-exposed mouse ear skin (Figure 3E). Secondly, rapid local release of endogenous NO from sinusoidal capillary vessels in response to tumor cell arrest has tumoricidal and antimetastatic effects [29]. Lastly, exogenous NO by oral NO-aspirin (NCX-4016) was demonstrated to potentiate antitumor immunity by reversing the immune-suppressing inflammatory effects of the myeloid cells in tumor-bearing mice [30]. Collectively, these findings further support the previous suggestion that stimulation of endothelial NO generation maybe an important contributing mechanism behind the reported antimetastatic benefits of certain β-blockers against cancer via normalization of anticancer immunity [10]. Furthermore, the same idea maybe extended in explaining the antimetastatic effects of other compounds that stimulate endothelial NO generation with immunity normalizing effects such as metformin, aspirin and other non-steroidal antiinflammatory drugs (NSAIDS) (Table 1) [31–36].

**Table 1:** Pharmacological characteristics of some of the reported antimetastatic drugs concerning their capacity to induce endothelial NO production and normalize immunity

Agent	Endothelial NO release stimulation	Immunity normalizing effect	Direct tumor cytotoxicity	Antimetastatic activity	Cancer risk reduction	NO release stimulation mechanism
Propranolol	yes [41]	yes [42]	no	yes [4]	yes [43]	NO-cGMP pathway activation [41]
Aspirin	yes [33]	yes (review: see ref. [31])	no	yes [44]	yes [44]	NOS3 acetylation [33]
Metformin	yes [35]	yes [34]	no	yes [36]	yes [45]	Direct donation [35]

The idea that the endothelial NO-release stimulation may be the main mechanism behind the immunity-mediated antimetastatic and cancer preventive benefits of certain β-blockers implicates several points of optimization for future studies in cancer treatment. In selecting the β-blocker for cancer therapeutic use, non-selective agents with notable endothelial NO-release stimulation activity such as carvedilol may be better candidates, although β1-selective agents with strong NO-releasing properties such as nebivolol or celiprolol may be interesting choices for investigation. However, in consideration of the fact that severe vascular dysfunction is reported in aged population or in those undergoing chemotherapies [37, 38], further supplementation with NO-donating agents such as metformin [35] or nitroglycerin may be additionally considered. Also, minimization of vascular toxicity from chemotherapeutic agents [37] needs to be considered by employing drug deliver system (DDS)-assisted agents such as Nab-paclitaxel since the vascular toxicity may impair endothelial NO-synthesis mechanisms. In support of this projection, the use of cytotoxins was clinically reported with increased incidence of metastasis development [39]. Thirdly, given the recommended use of DDS-assisted chemotherapeutic agents, co-administration collagen-degrading agents such as losartan or telmisartan may be considered for solid tumors *in vivo*. Collagen buildup surrounding most tumor vasculatures dramatically reduces drug penetration into cancer tissues, and hence administration of these agents enhances treatment effects by enhancing drug permeability through the collagen barrier [40]. Lastly, but most importantly, co-administration with immunotherapeutic agents is highly recommended as particular synergism between immunotherapeutics and NO-releasing agents is reported in animal model studies. In an animal model study using immunocompetent F344 rats, surgery stress suppressed the antimetastatic effects of NK cell immunostimulation by IL-12, which was fully restored upon nadolol/indomethacin treatment [9]. Similarly, NO-specific potentiation of cancer vaccine treatment effect was also reported in an immunocompetent Balb/c and C57BL/6 mouse model study using NO-aspirin (NCX-4016) [30]. Collective incorporation of these considerations may enable effective control of metastasis and tumor recurrence, which are among the major causes of cancer treatment failures. Henceforth, future clinical and preclinical studies investigating combinational use of NO-releasing β-blockers, immunotherapeutics, DDS-assisted cytotoxins and anticollagenic agents such as telmisartan against *in vivo* cancer are warranted.

## MATERIALS AND METHODS

### Materials

All chemicals used in this study were purchased from Sigma Aldrich (Saint Louse, MO) or SantaCruz Biotech (Dallas, TX). Antibodies were purchased from BD Biosciences (Franklin Lakes, NJ). Female Balb/c mice at 5 wk were purchased from Koatech (Korea). All experiments involving animals were performed in accordance with relevant guidelines and regulations of Korean authority, POSTECH (Pohang, Korea), and Vaccine Institute (Seoul, Korea). No blinding of animal subjects was needed in this study due to their gender and age uniformity.

### Characterization of inflammation in Balb/c mouse ears upon topical TPA and/or drug treatments

5 wk-old female Balb/c mice were ordered and stabilized for a week prior to the experiment. All drug treatments were given in 12 μL volumes. Each mouse ear was treated with acetone vehicle or 0.4 nmol TPA in acetone. These treatments were preceded with or without 1 nmol ATRA, 2 nmol pindolol, 2.5 nmol heptaminol, 2.2 nmol verapamil, or 1 nmol cPTIO by 30 minutes. For 24 h after the drug treatments, the ears were studied by TPM using live animals, or collected at ear root for analysis by hematoxylin & eosin-stained (H&E) histology or flow cytometry. Mouse ears showing obvious signs of self-inflicted injuries were excluded. Live TPM imaging of the mouse ears were performed using Leica two-photon microscope (TCS SP5II MP, Leica Microscopy Systems, GMBh) and a tunable Ti-Sapphire laser (Chameleon Vision II, Coherent) setup at 780 nm with a 20x water immersion objective lens (HCX IRAPO L20x, NA 1.0W, Leica). The live images were captured using 4 NDD detectors set up at 457/50 nm, 525/50 nm, 585/40 nm, and 650/50 nm. The TPM image data were processed using LAS AF Lite (Leica). In order to avoid artifacts from thrombosis due to excessive photo-exposure, rigorous measures were taken to avoid the artifacts from photo damage including thorough comparison with control samples under the similar exposure. For H&E-stained histology analyses, fresh samples were first fixed in 4% paraformaldehyde at 4°C overnight, which were then embedded in paraffin for sectioning at 8 μm thickness. These sections were then mounted onto slides and deparaffinized for H&E staining and observation under bright field microscope or TPM. Epidermal and skin thicknesses were manually measured from these histological images using calibrated rulers. 15 thickness readings were taken from each slide image and averaged. Three slides were chosen from each animal sample, and three biological replications were performed (*n* = 3 × 3 = 9).

For flow cytometry analysis, cells were isolated from the ear samples using the previously described method for studying ear skin-marginating NK cells [22]. Briefly, the collected mouse ears were split into dorsal and ventral sides, then briefly washed in RPMI buffer supplemented with 10% FBS. For each sample, 2 mouse ears were digested using collagenase IV (154 U/mL) in 10% FBS/RPMI buffer at 37°C for 2 h, followed by a dissociation through 100 μm cell strainer. The collected cells were washed and labeled with the following antibodies using BSA as the blocking agent against non-specific binding: CD45-PerCP-Cy5.5 (BD Pharminogen^TM^: 550994), CD3 Molecular Complex-FITC (BD Pharminogen^TM^: 555274), CD335 (NKp46)-Alexa Flour 647 (BD Pharminogen^TM^: 560755), TCR γδ-PE (BD Pharminogen^TM^: 553178). Meanwhile 7-AAD was used to assess cell viabilities (BD Via-Probe^TM^: 555816). The stained cells from 2 entire ears were characterized up to 100,000 events on BD Accuri^TM^ 6 Cytometer and its Software (BD Biosciences). The gating conditions were adapted from control experiments on spleen cells.

### Statistics

Two-sided student's *t*-test was used when comparing two values of an experiment. *P* values and *n* values are given in the figure legends or main text. Minimum number of animals needed for each study was determined by one-sided power analysis for mean differences from preliminary studies at power of 0.80. Accordingly three animals per treatment conditions were used in this study.

## SUPPLEMENTARY MATERIALS


