# Supplementary material for: NO-dependent attenuation of TPA-induced immunoinflammatory skin changes in Balb/c mice by pindolol, heptaminol or ATRA, but not by verapamil
Source: Oncotarget. 2016 Jun 22;7(30):47576–85. doi: 10.18632/oncotarget.10217 (PMC5216962; doi:10.18632/oncotarget.10217)
Supplement: Supplementary file 1 [file oncotarget-07-47576-s001.pdf]

NO-dependent attenuation of TPA-induced immunoinflammatory skin changes in Balb/c mice by pindolol, heptaminol or ATRA, but not by verapamil

Supplementary Materials

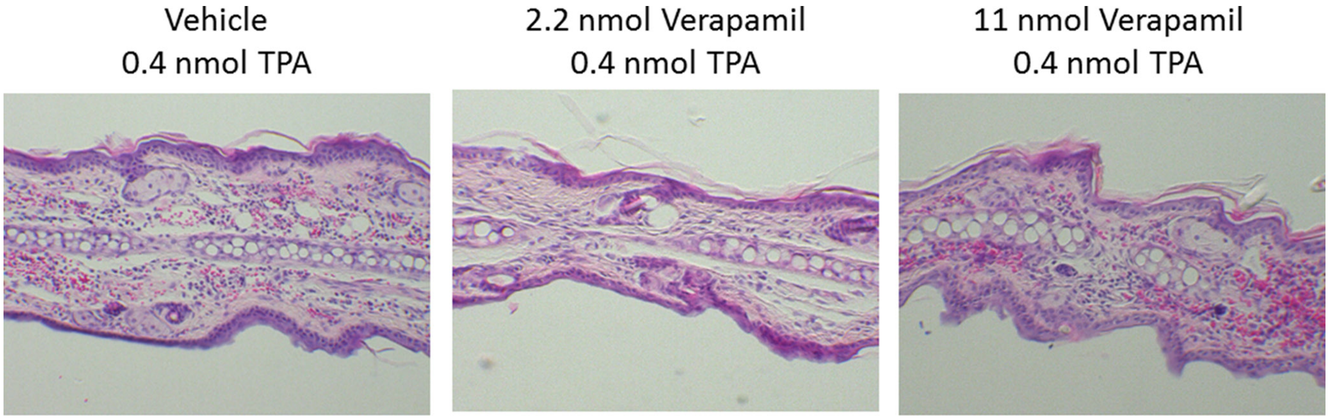

Criteria	TPA	TPA+Ver1x	TPA+Ver5X
Epidermal cell status	Atypical	Atypical	Atypical
Epidermal hyperplasia	+++	++	+++
Leukocyte Infiltration	+++	++	++++
Fibrosis	Present	Present	Present
Vascular leakage	+++	-	++++

Supplementary Figure S1: Increasing the verapamil dose does not help attenuate TPA-induced inflammation, hyperplastic changes, nor the ear skin tissue damage.
